# Structural and Morphological Features of Anisotropic Chitosan Hydrogels Obtained by Ion-Induced Neutralization in a Triethanolamine Medium

**DOI:** 10.3390/gels9110876

**Published:** 2023-11-04

**Authors:** Sergei L. Shmakov, Tatiana S. Babicheva, Valentina A. Kurochkina, Tatiana N. Lugovitskaya, Anna B. Shipovskaya

**Affiliations:** 1 Chair of Polymers, Institute of Chemistry, Saratov State University, 83 Astrakhanskaya St., 410012 Saratov, Russiashipovskayaab@yandex.ru (A.B.S.); 2 Institute of New Materials and Technologies, Ural Federal State University, 19 Mira St., 620002 Yekaterinburg, Russia; t.n.lugovitskaia@urfu.ru

**Keywords:** chitosan, anisotropic hydrogels, glycolic acid, triethanolamine, Liesegang structures, biocompatible polymers

## Abstract

For the first time, anisotropic hydrogel material with a highly oriented structure was obtained by the chemical reaction of polymer-analogous transformation of chitosan glycolate—chitosan base using triethanolamine (TEA) as a neutralizing reagent. Tangential bands or concentric rings, depending on the reaction conditions, represent the structural anisotropy of the hydrogel. The formation kinetics and the ratio of the positions of these periodic structures are described by the Liesegang regularities. Detailed information about the bands is given (formation time, coordinate, width, height, and formation rate). The supramolecular ordering anisotropy of the resulting material was evaluated both by the number of Liesegang bands (up to 16) and by the average values of the TEA diffusion coefficient ((15–153) × 10^−10^ and (4–33) × 10^−10^ m^2^/s), corresponding to the initial and final phase of the experiment, respectively. The minimum chitosan concentration required to form a spatial gel network and, accordingly, a layered anisotropic structure was estimated as 1.5 g/dL. Morphological features of the structural anisotropic ordering of chitosan Liesegang structures are visualized by scanning electron microscopy. The hemocompatibility of the material obtained was tested, and its high sorption–desorption properties were evaluated using the example of loading–release of cholecalciferol (loading degree ~35–45%, 100% desorption within 25–28 h), which was observed for a hydrophobic substance inside a chitosan-based material for the first time.

## 1. Introduction

Layered spatially oriented chitosan-containing materials, which have been intensely studied recently, are promising for use in tissue engineering (bone tissue replacement), regenerative medicine (a temporary construct for restoring epithelial and connective tissue), and also for the development of controlled drug delivery systems [1,2,3,4,5]. Such materials are of particular importance to manufacture scaffolds [6,7,8,9]. The layered structure provides high porosity, which is important for biomedical applications [2,10]. To obtain layered materials, intermittent precipitation reactions are used, among others, leading to the formation of Liesegang structures. A feature of the process of their preparation is the layer-by-layer periodic nature of the interaction of the salt chitosan form ([~-NH_3_]^+^) with alkali during the reaction of the polymer-analogous salt → base transformation, accompanied by deprotonation (neutralization) of the polycation to form a water-insoluble chitosan basic form ([~-NH_2_]↓) [6,11]. As a result, the formation of numerous intra- and intermolecular hydrogen bonds in macrochains, the occurrence of hydrophobic interactions, and the formation of chitosan base crystallites proceed in the system cyclically rather than simultaneously, in a local layer space each time. This makes it possible to generate a unique polymeric structure with spatially separated layers. A noteworthy feature is that chitosan in such a complex process not only performs the function of a highly viscous supporting medium but also serves as one of the reacting components (a carrier of functional groups).

The described method can be used in various ways, e.g., layer-oriented hydrogel materials were obtained by ion-induced precipitation of chitosan acetate in highly concentrated alkali media (5–10% NaOH) [6,12,13]. A solution of the salt chitosan form was either placed into a mold of one configuration or another with a hole at the top, specifying the desired architecture of the resulting product, and kept in an alkaline medium [6], or subjected to repeated alternation of neutralization of protonated amino groups and soaking in water [12]. The electrodeposition of chitosan chloride onto a steel wire by means of time-controlled charge transfer was also used [13] to form hydrogels with a similar periodic structure. In all these cases, the quantitative characteristics of the formation of periodic layer structures are consistent with the Liesegang phenomenon, and the layers were oriented along the diffusion front of hydroxide ions. It is also noteworthy that the formation of such multilayer hydrogels requires no use of auxiliary crosslinking agents and, therefore, avoids the formation of any side products of their condensation with chitosan macrochains during the formation of spatially crosslinked structures. When a chitosan acetate solution (with the addition of inorganic salts) was injected into an alkali solution through a circular nozzle [14], tubes with folded walls were formed. The authors believe that such bioinspired materials with multilayer supramolecular ordering are promising for use as thin films in fuel cells and lithium-ion batteries. Considering the biological activity of chitosan, it can be assumed that such microtubular substrates could be very promising in medicine, in particular, for prosthetics of blood vessels. It should be noted that a significant excess of the NaOH solution over the chitosan one by volume has always been a prerequisite for the formation of Liesegang hydrogel structures [6,12,13,14].

The engineering of cartilage tissue is another interesting area of practical use of hydrogels with spatially layered supramolecular ordering such as Liesegang structures. Runser et al. used enzyme-assisted self-assembly of peptides, organizing diffusion of phosphorylated peptides into a phosphatase-functionalized host gel [15]. The reaction–diffusion processes proceeding made it possible to obtain self-assembled structures with at least two self-assembly maxima. Self-assembling peptide hydrogels mimic the natural extracellular matrix and allow cells to grow, proliferate, and differentiate [16]. In addition, such biomimetic peptide hydrogels may exhibit orientated periodic ordering not only on a macroscopic but also on a microscopic scale, as was found in a study of diffusion-limited crystallization of calcium phosphate in a gelatin hydrogel [17]. All of the above opens up additional opportunities for structural diversification of chitosan-containing hydrogel materials with an anisotropic layered periodic supramolecular structure in biomedical applications.

Earlier, we studied the formation of layered ordered structures using a chitosan solution in glycolic acid and a strong alkali solution (5% NaOH) as a neutralizing agent [18], which is traditionally used to precipitate chitosan from aqueous acidic solutions. The process was carried out with no use of molds, i.e., without limiting the volume of the reaction system, and the volume ratio of NaOH: chitosan solutions did not exceed 1:1. The chitosan base was formed as swollen films with a water content of ~95% (so-called hydrofilms), which from two to seven Liesegang bands were observed on, depending on the polymer concentration (the dependence of the number of bands on the alkali concentration was not studied, but its existence could be assumed). Depending on the preparation method, the morphology of such gel films was represented either by a regular 1D sequence of parallel bands 1–36 mm wide (macroscopic scale) or by ring spherulites and an array of spatially separated microrings of circular symmetry, whose width was ~2–6 μm and increased from the center to the periphery (microscopic scale). The average diffusion coefficients of hydroxide ions in these periodic formations were (6.7–17.4) × 10^−10^ m^2^/s, increasing with the chitosan glycolate concentration in solution. In each experiment, only one value of this quantity was obtained (the experimental points fell on one straight line, see below). The reaction rate constant was equal to k = 0.48 ± 0.01 L/(mol∙min), and the ratio X_n+1_/X_n_ between the coordinates of adjacent bands was ~1.05.

It can also be assumed that the achieved number of bands depends on the nature of the neutralizing agent and on the diffusion coefficient of the molecules of this reagent in the medium of a viscous solution and/or hydrogel. It follows from general considerations that the diffusion rate of the reagent molecules and, consequently, the rate of the ionic reaction with the seizure of the polymer into a gel do not allow the formation of such structures that could appear during a slower neutralization reaction. Such structures could have more Liesegang bands and therefore be more anisotropic porous and more valuable for a number of biomedical applications. Therefore, it is advisable to study the use of other neutralizing (basic) agents, in particular, hydrophilic organic bases with neutral molecules competing with the amino groups of chitosan for a proton.

In addition, the use of strong alkali is somewhat problematic from the viewpoint of further practical application of the materials obtained, since it is harmful to biological tissues, and it is difficult to extract it completely from the material. Therefore, it would be preferable to use a soft organic base, preferably a pharmacopoeial one, whose traces remaining in the material cannot harm the body.

We chose TEA for this purpose, since it is highly soluble in water, approved for use in medicine, and its pKa (7.74 [19]) significantly exceeds this value for chitosan amino groups (6.0–6.3, according to various sources), which is the condition of effective deprotonation of these groups. In recent years, it has been increasingly used for deprotonation of the salt form of chitosan [20,21,22]. It should be noted that whereas a strong inorganic alkali directly dissociates into OH^-^ ions and a cation, TEA first interacts with a water molecule and is protonated, releasing a hydroxide ion into the solution. The latter, due to its relatively small size and high mobility, more easily penetrates the protonated amino groups of chitosan located inside the macromolecular coil than the TEA molecule could itself. When the structure of the chitosan material is compacted, it can be expected that OH^-^ ions can predominantly pass through it. Our first results showed that the hydrogel objects obtained by neutralization in the TEA medium differ significantly from those obtained in the NaOH one in their morphostructure and anisotropy of the supramolecular ordering of the polymer material [23].

Since the main applications of layered chitosan-containing materials are in medicine and pharmaceuticals [1,2,3,7,10,24], glycolic acid, as earlier [18], was used to convert chitosan into its salt form. The rejection of classical aqueous acidic solvent media (acetic and hydrochloric acids) was motivated not only by the pharmacological activity of this hydroxy acid but also by the previously established kinetic stability of chitosan glycolate hydrogels [25,26]. It is also known that the grafted composite of chitosan–glycolic acid with gold nanoparticles is biocompatible, stable over a wide pH range, and has a highly developed porous structure and a satisfactory drug release rate [27].

It is customary to test newly obtained chitosan-containing materials for sorption–desorption of drugs, and, due to the nature of chitosan, mainly hydrophilic drugs were taken [28,29,30,31]. In the case of creating materials for the controlled delivery of hydrophobic drugs, a number of objective difficulties are noted [32], e.g., in some studies of the sorption of nonpolar hydrophobic cholecalciferol (vitamin D_3_) by nanoparticles based on chitosan-containing complexes [33,34,35], a number of difficulties were noted both in loading this drug and in maintaining its bioavailability. We have hypothesized that the layered structure with a large number of Liesegang rings is capable of retaining and sustaining the release of such hydrophobic drugs, expanding the scope of the application of layered materials in medicine. Therefore, we chose cholecalciferol to test the obtained chitosan films as a potential drug delivery agent. It is highly soluble in non-polar solvents, and its solubility in water is less than 100 mg/L at room temperature. The biological effect of vitamin D_3_ in the human body is determined by calcium regulation, as well as the modulation of cell growth, neuromuscular conduction, and immunity [36]. The total intake of vitamin D_3_ in an adult should not exceed 2000 IU per day [37].

The aim of this work was to study the formation of Liesegang structures during the reaction of polymer-analogous transformation of chitosan glycolate → chitosan upon interaction with TEA, to evaluate quantitative characteristics of the mass transfer of this process, and to assess the samples obtained as sorbents of drugs using cholecalciferol as an example.

## 2. Results and Discussion

### 2.1. Viscosity Properties

Practice shows that the acid anion has a strong (sometimes decisive) effect on the properties of solutions, gels, and materials obtained from chitosan [2,6,12,13,14,15,18,25,26]. In this regard, chitosan in an aqueous acidic solution should be defined as a salt of one or another acid; in our case, when the polymer is dissolved in an aqueous solution of GA, chitosan glycolate is formed. However, it should be noted that such a nomenclature is arbitrary, since the stoichiometric chitosan:acid ratio, as a rule, is not observed, since when chitosan is dissolved, even in an excess of acid, the degree of protonation of –NH_2_ groups is less than 1 (rel. units) [18]. In the future, the term “chitosan glycolate” will be applied to a polymer solution in glycolic acid and gel film materials obtained therefrom; however, the concentration will be indicated only for chitosan, ignoring the acid anion.

Since the reaction of deprotonation of the amino groups of chitosan with an organic base should proceed more slowly than in the case of strong alkali, the proper choice of the concentration of the chitosan salt solution is of particular importance. When the amino groups of the chitosan macromolecule are deprived of protons and become neutral, the chain gradually ceases to be stretched due to the weakening of the electrostatic repulsion and folds into a coil. If this happens before sufficiently strong hydrogen bonds with neighboring macromolecules are established, the formation of a 3D gel network will become problematic. This is caused, first of all, by the required solution viscosity, whose minimum value is determined by the critical concentration of the polymer. Therefore, it was necessary to evaluate the critical concentration of entanglements for chitosan glycolate and choose the proper working values of the polymer concentration with some margin.

**Figure 1:**
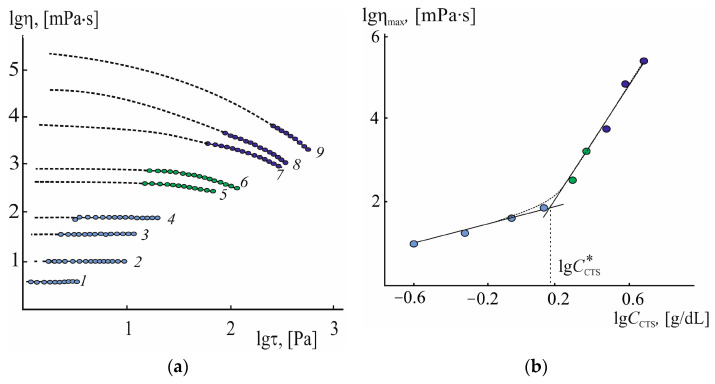
(**a**) Shear viscosity rheograms of chitosan glycolate solutions with *C*_CTS_ = 0.25 (*1*), 0.5 (*2*), 1.0 (*3*), 1.5 (*4*), 2.0 (*5*), 2.5 (*6*), 3.5 (*7*), 4.0 (*8*) and 4.5 g/dL (*9*); round markers—experimental data; dotted lines—calculation by the Vinogradov–Pokrovsky model [36], blue circles correspond to Newtonian flow, green ones – to Newtonian and structural viscosity, and lilac ones – to structural viscosity; (**b**) concentration dependence of the highest Newtonian viscosity in double logarithmic coordinates for the same solutions of chitosan glycolate as in (**a**), $C_{\mathrm{CTS}}^{*}$ is the polymer concentration during the formation of a fluctuating network of entanglements (1.5 g/dL).

Figure 1a shows the shear viscosity rheograms of the solutions studied. It can be seen from the figure that the viscosity of solutions of relatively low concentrations (*C*_CTS_ ≤ 1.5 g/dL) does not depend on the shear stress and the flow curves are described by almost linear dependences lgη = *f*(lg τ) (lines 1–4). With an increase in the polymer concentration and, accordingly, a decrease in the molar polymer:acid ratio, the viscosity of the solution increases, and the nature of its flow changes, e.g., for moderately concentrated solutions with *C*_CTS_ = 2.0–2.5 g/dL, flow curves are observed typical of polymer systems with regions of the highest Newtonian and structural viscosities (curves 5 and 6). When *C*_CTS_ ≥ 3.5 g/dL, the solutions exhibited abnormally viscous flow with a region of structural viscosity; their viscosity decreased sharply with an increase in shear stress over the entire range of the latter (curves 7–9), which is typical for the flow of pseudoplastic systems.

The highest Newtonian viscosity of chitosan solutions was estimated from the viscosity rheograms. For low-concentrated solutions, the viscosity readings began with it, but the device did not allow applying low mechanical stresses for high-concentrated viscous solutions, and the dotted lines in Figure 1a show the course of rheograms in the region of low stresses (calculation according to the modified Vinogradov–Pokrovsky model [38]). To fix the change in the mass transfer mechanism due to the formation of a physical network of macromolecular entanglements, we plotted the concentration dependence of the highest Newtonian viscosity (in double logarithmic coordinates) (Figure 1b). The concentration dependence of the highest Newtonian viscosity turned out to be classical for polyelectrolyte solutions with two rectilinear sections and a sufficiently extended intermediate curvilinear region in the middle. In the first straight section, the increase in the viscosity of chitosan glycolate solutions was almost proportional to the increase in the polymer concentration in the solution. Accordingly, within this concentration range, the chitosan-containing system does not meet the necessary conditions for the formation of a gel therefrom. As the polymer concentration in the system increased (in the second section of the concentration dependence), the highest Newtonian viscosity sharply rose, which indicated a change in the mass transfer mechanism and the formation of a physical network of macromolecule entanglements. $C_{\mathrm{CTS}}^{*}$ = 1.5 g/dL was found from the intersection point of the two straight sections as the minimum concentration of chitosan required to form a 3D gel network. Based on this, the working concentrations of the polymer were chosen not less than 2 g/dL, but not higher than 4.5 g/dL, since it was not possible to obtain them at the used concentration of GA.

### 2.2. Appearance

**Scheme 1:**
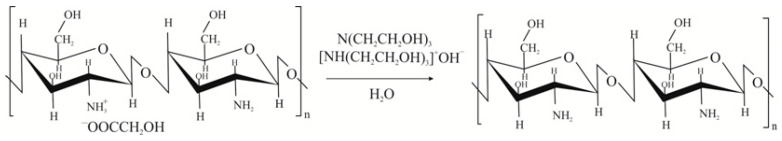
Reaction of polymer-analogous conversion of chitosan glycolate–chitosan base using TEA as a neutralizing agent.

At the next stage, gel films were obtained by the polymer-analogous transformation of chitosan glycolate → chitosan base in a water–TEA medium (Scheme 1). Our estimated calculation showed that at a 50% concentration of TEA, the vast majority of it is in the form of neutral molecules; therefore, the refractive index of the solution depends on the concentration of the uncharged form and makes it possible to monitor its diffusion through the interface. Hydroxide ions play an auxiliary role, participating in the acid–base equilibrium and deprotonation of the amino groups of chitosan.

**Figure 2:**
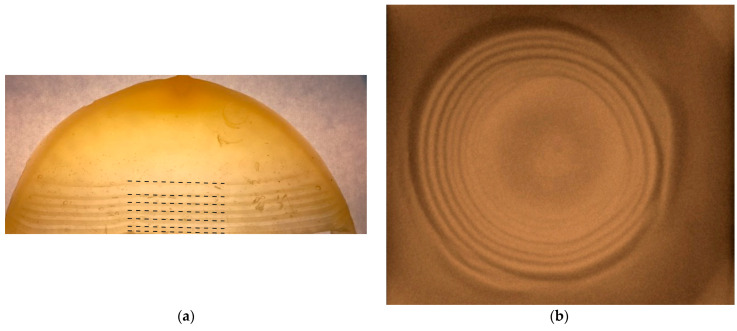
Photo of a gel film with a tangential periodic structure obtained by reacting 4 g/dL chitosan glycolate solution with TEA (“boundary by diameter” (**a**) and “diffusion from center” (**b**)). Dashed lines are drawn to improve the contrast of the bands.

Figure 2 shows photos of a typical chitosan gel film obtained by the “boundary by diameter” and “diffusion from center” method in the bulk of a flat layer of a chitosan glycolate solution after the salt → base chemical reaction. TEA molecules (as well as their accompanying hydroxide ions) diffused from the diameter or from the center (solution contact boundary) to the periphery of the Petri dish. In the first case, the reaction proceeds at the same volume ratio (the solution of the salt polymer form—the neutralizing reagent solution) and only with horizontal diffusion, while in the second case, it goes in the deficiency of the neutralizing reagent which diffuses vertically as well as horizontally. The solid phase of basic chitosan was formed as a banded periodic structure or concentric rings with 3D plane parallel zones (they are marked with dotted lines in Figure 2).

Visually observed distinct bands appeared mainly in the first hours of the experiment. Detailed information about the bands (formation time, coordinate, width, height, and formation rate) is given in Table 1.

The following formulae were used to calculate S. The area of a segment of a circle with radius R is expressed by the formula $\;S=\frac{R^{2}}{2}\left(\theta -\sin\theta \right)$, where θ is the angle at which the chord cutting off the given segment is visible from the center of the circle. Accordingly, the area of the layer between the diameter of the circle and the chord parallel to it, spaced from it by a distance L (band width ΔX_n_), is equal to $S=R^{2}\arcsin\frac{L}{R}+L\sqrt{R^{2}-L^{2}.}$

### 2.3. Liesegang Patterns

**Figure 3:**
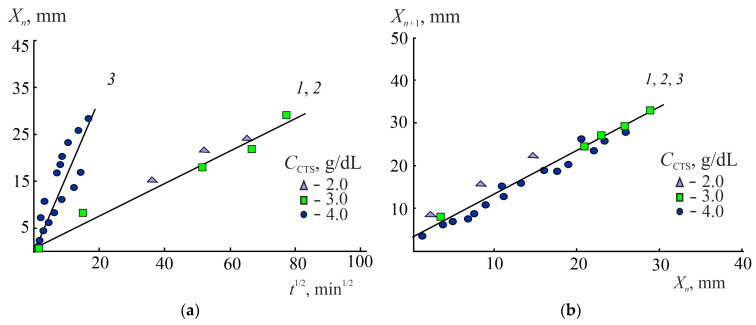
(**a**) Dependence of the distance *X*_n_ from the interface to the *n*th Liesegang band on the square root of time; (**b**) ratio of the positions/coordinates of neighboring *X*_n_ and *X*_n+1_ bands during the formation of a chitosan gel film (“boundary by diameter”) using TEA as a neutralizing agent; *C*_CTS_ = 2 (1 ∆), 3 (2 □), and 4 g/dL (3 ○).

On the graphs in the Liesegang coordinates (Figure 3a,b), rectilinear dependences *X*_n_~$\sqrt{t_{n}}$ and *X*_n+1_~*X*_n_ are observed, and the ratio of the positions of adjacent periodic bands approaches a constant, close to unity: *X*_n+1_/*X*_n_ ≈ 1.01. Therefore, as in the case of using NaOH [18], the ratio of the positions of periodic bands and the kinetics of their formation are described by the law of time and space, which is characteristic of the Liesegang phenomenon [6,12,13]. What is new is that the number of bands in the case of using TEA was significantly greater and reached 16 for the highest concentration of chitosan (Table 1). It was this solution that had the maximum viscosity, and it was for this solution that the band formation time was the longest.

### 2.4. Kinetic Data

**Figure 4:**
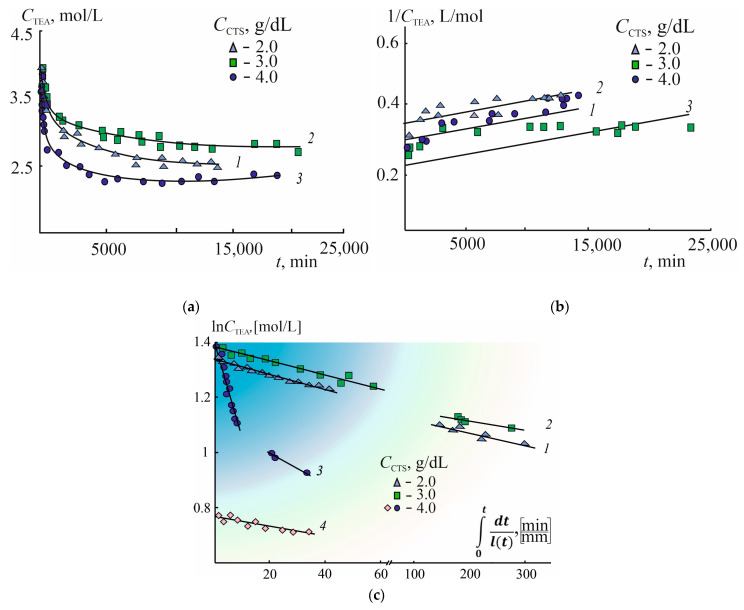
Change in the concentration of the neutralizing agent in the coordinates *C*_TEA_ vs. *t* (**a**), 1/*C*_TEA_ vs. *t* (**b**), and ln*C*_TEA_ vs. $\int_{0}^{t}\frac{dt}{l(t)}$ (**c**) during the reaction of polymer-analogous transformation chitosan salt (glycolate) → chitosan base (“boundary by diameter”) using TEA (1 ∆, 2 □, 3 ○) and NaOH (4 ◊ [18]) as a neutralizing reagent; *C*_CTS_ = 2 (1 ∆), 3 (2 □), and 4 g/dL (3 ○, 4 ◊).

In the course of the “boundary by diameter” experiment, the TEA concentration sharply decreased over the first day (Figure 4a) and then gradually reached a plateau. When a constant value of the TEA concentration was reached, the reaction of polymer-analogous transformation chitosan salt → chitosan base could be considered complete, which was also confirmed visually (a white gel film was formed). Compared to previous experiments using the NaOH solution [18], the process proceeded almost twice as slowly.

The same data plotted as *C*_TEA_^−1^ vs. *t* give satisfactory straight lines (Figure 4b). This means the second order of the reaction, if diffusion is neglected, and the reaction rate constant for all three concentrations of CTS turned out to be k = (8 × 10)^−6^ L/(mol∙min).

To estimate the diffusion coefficient of TEA molecules in the chitosan gel formed, a graph was plotted in the ln*C*_TEA_ vs. $\int_{0}^{t}\frac{dt}{l(t)}$ coordinates according to the calculation method described elsewhere [18] (Figure 4c, lines 1−3). For comparison (line 4) the data for the previous case (NaOH) were given. In this case, the points on the graph lie on two rectilinear segments with different slopes rather than on one straight line, somewhere there must be an inflection point (unfortunately, due to time constraints during the experiment, it was not possible to observe the appearance of such an inflection point). This can be explained as follows. Since areas with more and less high density of the material alternate in the layer, the diffusion coefficient should change cyclically with thickness, and the fact that the points still lie on one straight line indicates that the averaging of D over the entire layer is correct. Thus, the value of *D* can be taken out of the integral sign and estimated from the straight line slope.

However, the fact that after some time after the beginning of the experiment, the straight line segment has a different slope indicates that the possibility of correctly averaging the diffusion coefficient decreases with an increase in the number of rings. It is said in the “Calculation part” that, as a first approximation, the assumed smooth change in D across the layer was replaced by a step, namely: *D*_1_ in the first section and *D*_2_ in the second one, respectively.

The values of the diffusion coefficients in both sections are given in the last columns of Table 1. As expected, the values of *D* in the second section are noticeably smaller than in the first one (a different decimal order). This can be explained as follows. As the diffusion front moves deeper into the chitosan solution, the TEA concentration decreases, and the rate of deprotonation of chitosan amino groups decreases as well (according to the law of mass action). The transition of the polymer macromolecule from its stretched state to a coil is slowed down, and it has more time to search for the most energetically favorable conformation with many hydrogen bonds (intermolecular and intramolecular). But the more energetically favorable the structure, the denser it is. All this leads to spatial limitations in the mass transfer of substances and a decrease in the diffusion coefficient. It is quite possible that the gel film limits the diffusion of TEA molecules and allows only small OH^−^ anions (formed during the interaction of TEA with water) to pass through.

### 2.5. SEM Morphostructure Analysis

**Figure 5:**
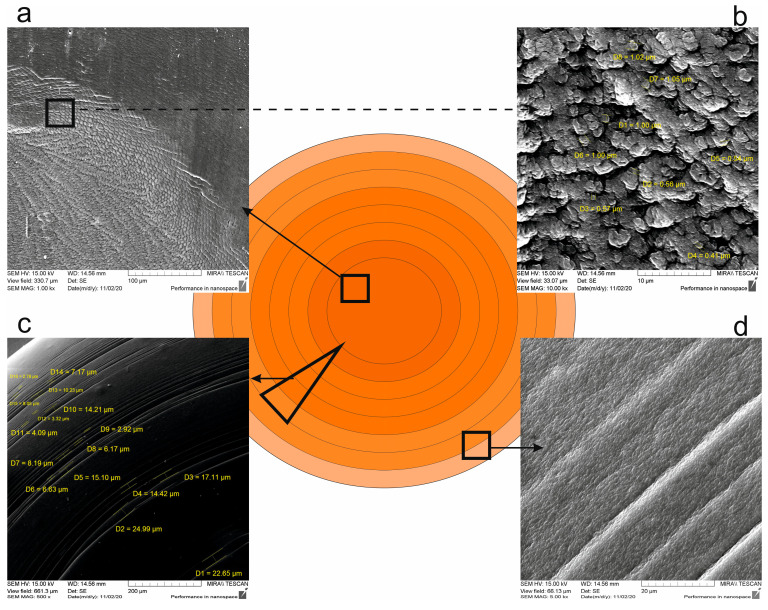
A schematic representation of our anisotropic hydrogel material (scheme at the center) and SEM photos of a gel film obtained by the reaction of a 4 g/dL chitosan glycolate solution with TEA (“diffusion from center”): (**a**,**b**) primary interface, (**c**) middle, and (**d**) peripheral concentric zones.

It is more convenient to study the morphology of the resulting structures on a microscopic scale on dehydrated gel films obtained by the other method (“diffusion from center”). Figure 5 shows SEM images of such films. It can be seen from the figure that the film morphology is represented by an array of microrings of circular symmetry, whose width varies in the range of 0.4–25 μm. As in the case of using NaOH [18], three zones are observed in these radial periodic structures, namely: a primary interface of an almost round shape with pronounced supramolecular ordering, which is formed at the initial stage of the interaction of the chitosan salt with TEA, middle concentric zones, and peripheral edge rings. A compaction of the material structure is observed from the center to the periphery.

Compared with the case of using NaOH [18], a number of significant differences were observed. First, the primary interface of the gel film obtained using TEA is represented by an ordered twist radial structure of granular supramolecular formations 5–15 µm wide rather than by ring spherulites (Figure 5a). Inside them, zones of spherulite-like scaly supramolecular aggregates are distinguished, resembling the granular microstructure of mineral bodies (e.g., concretions or geodes), separated by flat textures (Figure 5b). There is a characteristic feature, namely: the radial orientation of the granular configurations coincides with the direction of diffusion of the neutralizing reagent during the formation of basic chitosan. A similar radially ordered structure, only with lamellar supramolecular formations, was observed by Nie et al. during the ion-induced precipitation of chitosan acetate with a 10% NaOH solution in a cylindrical form [6]. Second, as the polymer-analogous transformation proceeds and moves away from the boundary of the beginning of the reaction to the periphery, the width of the rings decreases (Figure 5c). Third, a multilevel layered ordered architecture is observed at the periphery, and microrings 0.4–0.7 µm wide are found inside the ring formations (Figure 5d). For the first time, Jo et al. reported the inhomogeneity of an individual band in Liesegang patterns [17]. Our study supplements this fact with the example of another type of system, where the role of the hydrogel support matrix and the internal electrolyte is played by the same component, chitosan salt. Therefore, in comparison with NaOH, the kinetic features of the ion-exchange reaction in the absence of convection and mixing with the use of TEA are also reflected in the supramolecular ordering of the resulting gel films.

### 2.6. Functionality Studies

**Figure 6:**
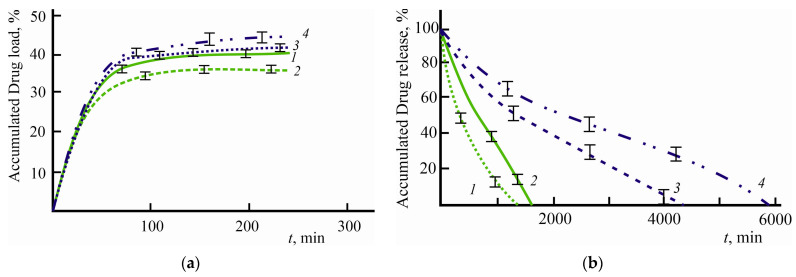
Kinetics of accumulated drug load of cholecalciferol during sorption (**a**) and accumulated drug release during desorption (**b**) from gel film samples obtained by the “boundary by diameter” method from a chitosan glycolate solution with *C*_CTS_ = 3 (1, 3) and 4 g/dL (2, 4) using TEA (1, 2, green lines) and NaOH (3, 4, lilac lines) as a neutralizing agent.

To assess the functionality of the layer-oriented structure of our chitosan gel films, at the next stage, the sorption–diffusion properties of the samples were studied in a model experiment of a drug delivery system. It turned out that the load degree of cholecalciferol into the layered chitosan matrix under our conditions can reach ~35–45% (Figure 6a, curves 1 and 2). A constant value of the degree of loading was achieved after 2 h of keeping the sample in the model solution. The duration of sorbate desorption from the polymer matrix was always longer than that of sorption (Figure 6b, curves 1 and 2). A 100% desorption was observed, on average, within 25–28 h. The chitosan concentration in the initial solution used to obtain the gel film affects both the quantitative characteristics and kinetics of the loading–release processes, e.g., an increase in the polymer concentration leads to the formation of a denser film with a large number of layers (see Table 1), which somewhat reduces, although not very significantly, the amount of sorbed vitamin *D*_3_. On the other hand, with an increase in the chitosan concentration in the initial solution, the ability of the gel film for prolonged release from the polymer matrix of the optimal drug concentration significantly improves since the total desorption time of cholecalciferol increases. Gel films obtained under similar conditions with the use of NaOH (for comparison) sorbed this drug with almost the same efficiency (Figure 6a, curves 3 and 4). However, the release of the drug lasted much longer, 72–100 h (Figure 6b, curves 3 and 4), and the concentration of released cholecalciferol was lower than the required (for optimal biological action) daily dose [37].

Thus, the layered structures obtained by us provide the ability to sorb not only well-studied hydrophilic substances but also hydrophobic ones (cholecalciferol). The use of gel films greatly simplifies any method of drug encapsulation into a hydrogel structure and, no less important, involves neither changes in its biologically active form nor thermal treatment of the polymer system. TEA could turn out to be preferable in comparison with NaOH as a substance allowed in pharmacopoeial practice, and also in the medium of which the layered structure of the chitosan gel film, which is optimal for controlled delivery of a pharmaceutical product in its desired concentration, is formed.

Our study of the general biological properties of the gel films showed they are hemocompatible. The hemolysis degree of human erythrocytes upon contact with samples obtained in the TEA medium was 4.0 ± 1.0%; that for NaOH (for comparison) was 1.6 ± 0.8%, which did not exceed that in physiological saline. Therefore, gel films made from neutralized chitosan glycolate can be used in biomedical and pharmaceutical applications, in particular, as blood contact materials.

## 3. Conclusions

In this work, by changing the nature of the reagent used to neutralize the chitosan salt in solution, anisotropic chitosan gel films with periodic Liesegang structures and radically increased porosity were obtained. Our assumptions were confirmed that under the action of a weak organic base (a TEA solution instead of a strong alkali one), a viscous solution of chitosan glycolate turns into a gel more slowly, the permeability of the resulting material for TEA molecules decreases gradually (however, the permeability for OH^−^ ions, apparently, remains), and the longer time interval before fixing certain sections of the material with gel crosslinks creates conditions for the emergence of a larger number of Liesegang rings/bands and the formation of a material with a developed layered supramolecular ordering. The complication of the processes occurring during neutralization is indirectly confirmed by the presence of two straight sections on the kinetic plot instead of one, as in the case of inorganic alkali. In addition, an essential advantage of the said organic base lies in its approval for use in the pharmacopeia, the resulting materials are hemocompatible, and the loading of a model drug (cholecalciferol) into them and its release were tested. Despite the hydrophilic nature of chitosan, the loading of the hydrophobic substance proceeded quite satisfactorily, which we attribute to the high porosity of our material. The peculiar layered porous structure of the gel film, apparently, also determines the optimal release of the drug in its recommended daily intake. The latter was not observed when NaOH was used as a neutralizing agent. We assume that in some cases the formation of Liesegang structures by the method described above could replace the laborious production of layered anisotropic materials by the layer-by-layer method [38], as well as the step-wise technique, photo-polymerization technique, and sequential electrospinning technique, which involve other polymers (alginate, cellulose, poly(ethylene oxide), polyε-caprolactone, poly(acrylic acid), poly(vinyl alcohol), poly(2-alkylacrylic acid), polyvinylpyrrolidone, poly(ethylene glycol), etc. [39]). It is to be expected that the directional layered orientation of the material would be beneficial in controlling drug transport and release at the intended site, increasing strength, and stopping crack propagation in bone and cartilage replicas (where layering is advantageous whilst solidity is more detrimental than beneficial), as well as in the design of other artificial prototypes of living systems.

## 4. Materials and Methods

### 4.1. Substances and Reagents

We used powdered chitosan (CTS) with an average viscosity molecular weight of 700 kDa and a deacetylation degree of 80 mol.% produced by Bioprogress Inc. (Moscow, Russia), 70% glycolic acid CH_2_(OH)COOH (GA) (Sigma-Aldrich, Saint Louis, MO, USA), TEA N(CH_2_CH_2_OH)_3_ (Baza No. 1 Khimreaktivov Inc., Moscow, Russia), sodium hydroxide NaOH (Khimreaktiv Inc., Moscow, Russia), sodium chloride NaCl (Vekton Inc., Moscow, Russia), sodium citrate Na_3_C_6_H_5_O_7_ (Vekton Inc., Novgorod, Russia). All reagents were chemically pure. Cholecalciferol C_27_H_44_O was used in the form of a pharmaceutical preparation of vitamin D_3_ DeTriFerol™ (Grotex Inc., Saint Petersburg, Russia) as a solution in propylene glycol: the content of the main substance was 15,000 IU (0.97 mmol/L).

### 4.2. Preparation of Solutions

A 1.5% (0.2 M) aqueous solution of GA, 50% aqueous solution of TEA (the optimal concentration was found in special experiments on the transfer of chitosan films to the basic form), 5% aqueous solution of NaOH, 0.9% (0.15 M) aqueous solution of NaCl, and 3.8% (0.147 M) solution of sodium citrate were prepared by dissolving the appropriate weighed portions (or aliquots) in distilled water at room temperature. Aqueous solutions of chitosan glycolate with concentrations *C*_CTS_ = 0.5–4.5 g/dL were prepared by dissolving an air-dry sample of polymer powder in a calculated volume of 0.2 M GA under stirring on a magnetic stirrer for ~2 (diluted) and 5–7 h (moderately concentrated). The latter ones were additionally kept for 24 h at room atmosphere to remove air bubbles.

### 4.3. Preparation of Chitosan-Based Gel Films

Gel films were obtained in two ways according to the techniques described elsewhere [18]. According to the first method, a Petri dish 8 cm in diameter was divided in diameter into two halves, which were filled with solutions of chitosan glycolate and an organic base, respectively (“boundary by diameter”), after the movement in liquids ceased, the partition was removed (the beginning of the reaction, *t* = 0). Obtaining objects by the second method included applying a base solution to the center of a Petri dish 6 cm in diameter filled with a polymer salt solution (“diffusion from center”). The height of the initial flat layer of the polymer solution was 5 mm in all cases. As a result of the chemical reaction of polymer-analogous transformation chitosan salt → chitosan base, which proceeded in the absence of convection and stirring at room temperature, a solid-phase water-insoluble chitosan-containing material of white color was formed, which included 87 ± 6% water. The resulting gel films were repeatedly washed with distilled water until a neutral pH value of the washing liquid. The presence/absence of impurities was controlled by the refractive index of the liquid medium in which the samples were stored.

### 4.4. Methods

Gravimetric measurements were carried out on an Ohaus Discovery analytical balance (Ohaus Corp., Parsippany, NJ, USA), with weighing accuracy being ±0.01 mg.

The water content in the samples was found on an A&D AND MS 50 moisture analyzer (A&D Ltd., Tokyo, Japan), the accuracy was ±0.01%.

Viscosity rheograms lg η = *f*(lg τ) were recorded on a Rheotest RN-4.1 rotational viscometer (Ottendorf-Okrilla, Germany) with a cylinder–cylinder operating unit (internal cylinder H1) in the shear stress range lg τ = 0.1–3.0 [Pa] at 25 °C, the preliminary thermostating time was 30 min. The values of the highest Newtonian viscosity η_max_ of concentrated solutions, which the initial region of the Newtonian flow was not experimentally fixed for (the device immediately set the values of τ which the destruction of supramolecular structures was already in progress at), were estimated using the modified Vinogradov–Pokrovsky rheological model and the MAXIMA program [40].

The refractive index $n_{D}^{25}$ was measured on a Mettler Tolledo RM-40 digital refractometer at 25 °C.

The dimensional characteristics of the periodic structures of our gel films were determined from digital photos using image analysis software; in some experiments, they were measured with an Electronic Digital Outside Micrometer (Kawasaki, Japan) with an accuracy of ±0.01 mm. Visual changes during the course of the chemical reaction were documented by digital photos taken with a Sony Alpha SLT-55 camera (Bangkok, Thailand) fixed in a tripod using overhead lighting.

### 4.5. Determining the TEA Concentration in the “Boundary by Diameter” Method

To find the decrease in the TEA concentration (∆*C*_TEA_) due to diffusion into the reaction area, an aliquot of the TEA solution with a volume of 1 mL was taken with an automatic dispenser, its refractive index $n_{D}^{25}$ was measured for 1–2 min and returned back to the system under study. Aliquots were taken at intervals of 5 min during the first hour of the experiment, then after 10 and 30 min during the next 2.5 and 7 h, respectively, and then after 24 h until the end of the experiment. The total reaction time was 40–120 h, depending on the chitosan concentration. The concentration of TEA was found according to a preliminarily constructed calibration curve $n_{D}^{25}$ = *f*(*C*_TEA_).

### 4.6. SEM Studying of Gel Films

The morphological structure of our gel films was examined by scanning electron microscopy (SEM) on a MIRA\\LMU microscope (Quorum Technologies Ltd., Ashford, Kent, UK) at a voltage of 15 kV and a conductive current of 400 pA. To prepare objects, a cubic mold 1 × 1 × 1 cm in size with removable sides was made from cover slips (manufactured by MiniMed, Bryansk, Russia) and filled with a chitosan glycolate solution. An amount of 250 μL (for 5 h, 50 μL every hour) of the TEA solution was applied to the center. The reaction product was dried in air at room temperature for 24 h. Prior to microscopic studies, a 5 nm thick layer of gold was deposited onto each sample using a K450X Carbon Coater (TESCAN GROUP, Brno–Kohoutovice, Czech Republic).

### 4.7. Sorption–Diffusion Experiment Technique

Drug loading and release were studied in a sorption experiment using freshly prepared chitosan gel films and a water–organic solution of cholecalciferol as a model drug. For sorption, a gel film as a circle with a diameter of 6 cm and a weight of 2 g (without drying, the humidity is indicated above) was placed into 2 mL of a sorbate solution (0.97 mmol/L cholecalciferol solution in propylene glycol) and kept on a PST 60HL thermoshaker (BioSan, Rīga, Latvia) at 350 rpm and 22 ± 2 °C for 24 h. The release of the sorbate from the polymer matrix was carried out in the desorption mode in 2 mL of distilled water, simulating biological fluids with a neutral pH value, under static conditions at room temperature. The cholecalciferol concentration in both cases was determined by the refractive index (see Section 2.5) using a pre-built calibration curve (dilution to create a series of concentrations was carried out with distilled water). It was assumed that the effect of excipients on the refractive index of the solution, as well as their sorption and desorption, can be neglected.

### 4.8. Hemocompatibility Assessment

Hemocompatibility was studied on a model of human erythrocytes by detecting oxidized forms of hemoglobin in vitro [41]. All solutions used in the experiment were pre-sterilized in an autoclave (Tuttnauer, Beit Shemesh, Israel) at 120 °C and a pressure of 0.11 MPa for 45 min. Samples of gel films with a surface area of 1 cm^2^ were sterilized in 70% ethanol for 20 min and kept in physiological saline for 24 h, placed in sterile Petri dishes (Falcon-BD, Miami, FL, USA), and a standard dose of erythrocyte suspension (optical density 0.5 ± 0.1 at 545 nm) was added and incubated on a BioSan PST 60HL thermoshaker (Rīga, Latvia) in shaking mode at 350 rpm and 37 °C for 1 h. Then the samples were centrifuged in a centrifuge shaker SM-70M-07 SIA (ELMI, Rīga, Latvia) at a rotation speed of 1000 rpm for 10 min and the optical density of the supernatant was measured on a Stat Fax 4200 photometer (Awareness Technology, Palm City, FL, USA) at λ = 545 nm. Three parallel experiments were carried out. The degree of hemolysis was calculated from the difference between the optical density of the supernatant of the test system and the erythrocyte suspension in physiological saline (positive control, 0% hemolysis), taking into account the optical density of the erythrocyte suspension in water (negative control, 100% hemolysis) and expressed as a percentage [42]. The test sample was considered hemocompatible if its degree of hemolysis did not exceed 5% [43]. Statistical data processing was carried out using Statistica 6.0.

### 4.9. Calculation Part

In our previous paper [18], an equation was derived for the Fickian diffusion of the base reagent in a Petri dish partitioned in half:

(1) $$\ln C\left(t\right)=\ln C\left(0\right)-\frac{DS}{V}\int_{0}^{t}\frac{dt}{l(t)},$$

where *C*(0) and *C*(*t*) are the concentrations of the base in the solution at the initial time and at time *t*, respectively, *l*(*t*) the layer thickness (distance from the partition to the diffusion front) as a function of time, *D* the diffusion coefficient of the base, *S* the cross-sectional area of the solution–gel film, *V* the volume of the base solution in the corresponding half of the Petri dish. The adequacy of this equation was confirmed by the fact that the experimental points in the coordinates ln*C*(*t*) vs. the integral fit well on one straight line, which made it possible to determine *DS*/*V*, and, consequently, the diffusion coefficient *D*.

In the present work (where the base was an aqueous TEA solution), the experimental points on the same graph fall not on one straight line, but on two rectilinear segments with different slopes, so the model should be modified. To describe the experimental results with an acceptable error, we assume that this equation is valid only in the first section (*t* < t*), where t* is the time at which, in the first approximation, an abrupt change in the diffusion coefficient of TEA is assumed. The assumed smooth change in *D* across the layer is replaced with a step: *D*_1_ and *D*_2_ in the first and second sections, respectively. In the second section, the integral was divided into two parts (because another value of the diffusion coefficient was taken out of the integral sign), and a new term appeared:

(2) $$\ln C\left(t\right)=\ln C\left(0\right)-\frac{D_{1}S}{V}\int_{0}^{t^{*}}\frac{dt}{l(t)}-\frac{D_{2}S}{V}\int_{t^{*}}^{t}\frac{dt}{l\left(t\right)}.$$

The fact that the layer thickness increases monotonically with time greatly simplifies the matter. The first two terms on the right-hand side (when we are already moving along the second section) are a constant value, which was the first term when moving along the first section; therefore, the coordinates of the graph are saved, and the possibility of estimating *D* by the slope coefficient also remains.

## Figures and Tables

**Table 1:** Spatio-temporal characteristics of tangential periodic structures in gel films obtained from chitosan glycolate solutions using TEA as a neutralizing agent, and physicochemical parameters of the polymer-analogous transformation chitosan salt → chitosan base occurring in this case (“boundary by diameter”).

Chitosan Solution Concentration *C*_CTS_, g/dL	Serial Number of the Band	Time *t* of Formation of the *n*th Band, min	Coordinate *X_n_* of the *n*th Band *, mm	Width ∆*X_n_* of the *n*th Band, mm	Height *h_n_* of the *n*th Band, mm	Volume Rate *U* of Formation of the *n*th Band **, mm^3^/min	Concentration ∆*C*_TEA_ of Reacted TEA, mol/L	TEA Diffusion Coefficient *D*·10^10^, m^2^/s	
								Staring Section	Ending Section
2	1	1.2 ± 0.7	2.7 ± 0.5	4.0 ± 0.5	4.7 ± 0.6	210 ± 60	0.1	16.8	5.41
	2	1400 ± 300	14.7 ± 0.5	10.5 ± 1.5	3.8 ± 0.4	2.3 ± 0.2	0.7		
	3	2600 ± 800	23.5 ± 0.5	11.0 ± 1.0	3.9 ± 0.5	1.3 ± 0.3	0.4		
3	1	1.5 ± 0.7	1.3 ± 1.1	0.5 ± 0.2	4.0 ± 0.8	28.3 ± 2.5	0.1	14.7	4.16
	2	4640 ± 40	17.6 ± 6.4	3.1 ± 1.2	4.8 ± 0.3	2.0 ± 0.1	1.0		
	3	4750 ± 80	23.6 ± 3.7	4.0 ± 0.5	4.8 ± 0.3	0.3 ± 0.1	1.1		
	4	4800 ± 800	29.5 ± 0.5	3.0 ± 0.5	4.8 ± 0.4	0.3 ± 0.1	1.1		
	5	7300 ± 500	32.5 ± 0.5	3.0 ± 0.6	5.1 ± 0.4	0.2 ± 0.1	1.2		
4	1	2.1 ± 0.1	1.3 ± 0.7	1.5 ± 0.5	2.2 ± 0.3	50.4 ± 7.9	0.1	153	32.9
	2	2.7 ± 0.4	3.0 ± 0.5	0.5 ± 0.2	2.2 ± 0.3	68.2 ± 10.3	0.1		
	3	4.3 ± 0.4	3.0 ± 0.5	1.0 ± 0.5	2.2 ± 0.3	37.1 ± 1.4	0.1		
	4	6.7 ± 2.2	4.0 ± 0.5	0.5 ± 0.2	2.2 ± 0.3	31.4 ± 8.1	0.1		
	5	10.0 ± 1.1	5.0 ± 0.5	1.0 ± 0.5	2.2 ± 0.3	19.0 ± 7.0	0.1		
	6	20.0 ± 6.7	6.0 ± 0.5	1.0 ± 0.5	2.3 ± 0.6	10.9 ± 2.0	0.3		
	7	40.0 ± 13	6.5 ± 0.5	0.5 ± 0.3	2.3 ± 0.6	10.9 ± 7.2	0.2		
	8	63.3 ± 10	7.0 ± 0.5	0.5 ± 0.3	2.6 ± 0.3	5.4 ± 2.2	0.2		
	9	80.0 ± 10	8.5 ± 0.5	1.5 ± 0.5	2.6 ± 0.3	4.7 ± 1.4	0.3		
	10	110 ± 10	11.5 ± 0.5	3.0 ± 0.5	2.7 ± 0.2	5.5 ± 2.3	0.4		
	11	170 ± 20	14.0 ± 0.5	2.5 ± 0.5	2.9 ± 0.3	3.3 ± 0.6	0.4		
	12	180 ± 20	15.5 ± 0.5	1.5 ± 0.5	3.2 ± 0.5	2.3 ± 1.1	0.8		
	13	210 ± 20	17.0 ± 0.5	1.5 ± 0.5	3.3 ± 0.4	2.5 ± 0.6	0.8		
	14	2700 ± 120	18.5 ± 0.5	1.5 ± 0.5	3.3 ± 0.4	1.9 ± 0.9	0.8		
	15	2760 ± 120	20.5 ± 0.5	2.0 ± 0.5	3.8 ± 0.5	1.9 ± 0.9	0.8		
	16	2800 ± 120	26.5 ± 0.5	6.0 ± 0.5	3.8 ± 0.5	1.6 ± 0.7	0.8		

* The coordinate of the *n*th band is the distance from the boundary of the beginning of the reaction to the position of the *n*th band. ** $U=\frac{S\cdot h}{t}$, where S is the interface area of the *n*th band of the chitosan gel film (mm^2^), h the average height of the *n*th band (mm), t the reaction time (min).

## Data Availability

Not applicable.
